# Cystamine and cysteamine as inhibitors of transglutaminase activity *in vivo*

**DOI:** 10.1042/BSR20180691

**Published:** 2018-09-05

**Authors:** Thomas M. Jeitner, John T. Pinto, Arthur J.L. Cooper

**Affiliations:** Department of Biochemistry and Molecular Biology, New York Medical College, Valhalla, NY, USA

**Keywords:** cystamine, cysteamine, celiac disease, cardiac disease, neurodegeneration, tranglutaminase

## Abstract

Cystamine is commonly used as a transglutaminase inhibitor. This disulphide undergoes reduction *in vivo* to the aminothiol compound, cysteamine. Thus, the mechanism by which cystamine inhibits transglutaminase activity *in vivo* could be due to either cystamine or cysteamine, which depends on the local redox environment. Cystamine inactivates transglutaminases by promoting the oxidation of two vicinal cysteine residues on the enzyme to an allosteric disulphide, whereas cysteamine acts as a competitive inhibitor for transamidation reactions catalyzed by this enzyme. The latter mechanism is likely to result in the formation of a unique biomarker, *N*-(γ-glutamyl)cysteamine that could serve to indicate how cyst(e)amine acts to inhibit transglutaminases inside cells and the body.

## Introduction

Cystamine is a symmetric organodisulphide commonly used as an inhibitor of transglutaminases. This disulphide is also reduced to cysteamine within the body. Cystamine and cysteamine both inhibit transglutaminases but by different mechanisms. Therefore, the purpose of this discussion is to highlight the redox behavior of cystamine and cysteamine *in vivo* and the mechanisms by which cystamine and cysteamine inhibit the activity of transglutaminases inside the body.

## Transglutaminases and the formation of cross-linked proteins in disease

**Figure 1:**
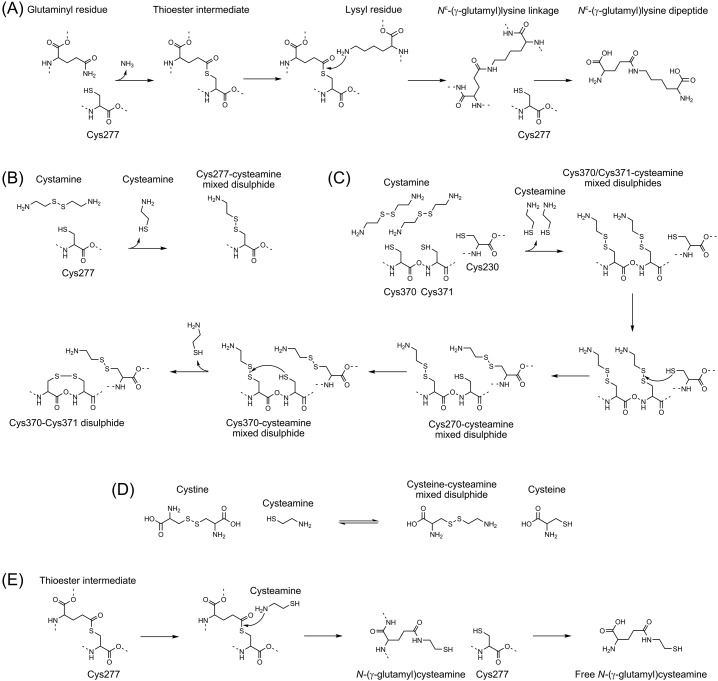
Reactions of cyst(e)amine with transglutaminases and cystine (**A**) Transglutaminase-catalyzed *N^ε^* (γ-glutamyl)lysine isodipeptide formation: transglutaminases catalyze an acyl transfer reaction that proceeds by a Bi-Molecular or Ping-Pong mechanism. Activated transglutaminases first act to form a thioester bond between the active site Cys^277^ and the carboxamide moiety of glutaminyl residues. Formation of this intermediate involves the release of the amide nitrogen as ammonia, which powers the subsequent catalysis. The thioester bond then undergoes a nucleophilic attack by the ε amine of lysine to complete the acyl transfer and produce *N^ε^* (γ-glutamyl)lysine isodipeptide linkage. These dipeptides can then be released from the protein by hydrolysis of the peptide linkages. (**B**) Oxidative inactivation of transglutaminase 2 by cystamine by the mechanism of Lorand and Conrad [46]: in this model, the thiol moiety of Cys^277^ participates in thiol-disulphide interchange with cystamine to produce cysteamine–Cys^277^ mixed disulphide. (**C**) Oxidative inactivation of transglutaminase 2 by cysteamine by our interpretation of the mechanism of Palanski and Khosla [48]: in this model, cystamine first forms mixed disulphides with Cys^370^ and Cys^371^. Cys^230^ then undergoes thiol–disulphide interchange with cysteamine–Cys^230^ mixed disulphide. The newly reduced Cys^371^ then reduces the mixed disulphide of cysteamine–Cys^370^ while being oxidized to the Cys^370^–Cys^371^ disulphide. It is also possible that the Cys^230^ undergoes thiol–disulphide interchange with the cysteamine–Cys^370^ mixed disulphide rather than the cysteamine–Cys^371^ mixed disulphide. In either case, the Cys^370^–Cys^371^ disulphide would form and allosterically regulate the enzyme. (**D**) Thiol–disulphide interchange of cysteamine and cystine: cysteamine interacts with cystine by thiol–disulphide interchange to from the cysteamine–cysteine mixed disulphide. Note that the latter resembles the lysyl residue depicted in (A). (**E**) Transglutaminase-catalyzed *N-*(γ-glutamyl)cysteamine formation: a mechanism for the competitive inhibition of transglutaminase by cysteamine. This mechanism is analogous to that shown in (A) and for the sake of brevity begins with thioester bound intermediate. The thio–ester bond is attacked by the amine nitrogen of cysteamine to complete the acyl transfer and produce *N-*(γ-glutamyl)cysteamine. We propose that *N-*(γ-glutamyl)cysteamine is released from the protein by proteolysis, as is the case for other *N*-(γ-glutamyl)amines.

Transglutaminases catalyze nucleophilic substitutions of the carboxamide group of glutaminyl residues [1,2]. The attacking nucleophiles are typically the amines of various compounds, but can include hydroxyl moieties and H_2_O depending on the transglutaminase isozyme or conditions. Thus, subject to the nucleophile, transglutaminases catalyze transamidation, esterification, or deamidation of glutaminyl residues. Transamidation involving the ε amine of lysyl residues is the reaction most often catalyzed by transglutaminases and results in the formation of *N^ε^*-(γ-glutamyl)lysine isodipeptide linkages between polypeptide chains (Figure 1A). A number of important pathologies exhibit both aberrant transglutaminase activity and increased production of *N^ε^*-(γ-glutamyl)lysine cross-linked proteins (*e.g.*, neurodegenerative disorders [3–12] and cardiovascular disease [13–20]). The involvement of increased transglutaminase activity in neurodegenerative or cardiovascular diseases is supported by the observation that genetic inactivation of various transglutaminases in animal models slows progression of these diseases [21–26]. The preceding observation and others prompted a search for medicinal transglutaminase inhibitors [27–29], as well as testing of cystamine in disease models and patients [30–45]. These tests indicate that cystamine might be of benefit in the treatment of selected diseases.

## Oxidative mechanisms for the inhibition of transglutaminases by cystamine

Cystamine was first reported to be an inhibitor of transglutaminase 2 by Lorand and Conrad in 1984 [46]. They hypothesized that cystamine and the active site Cys^277^ undergo a thiol-disulphide interchange to produce a mixed disulphide that prevents catalysis (Figure 1B). Cystamine is the disulphide form of cysteamine. Thus, the proposed thiol-disulphide interchange (thiolation) produces free cysteamine and a mixed disulphide of cysteamine and Cys^277^. An ‘oxidative mechanism’ for the inhibition of transglutaminase 2 is supported by subsequent investigations by Jeon *et al*. [47], and also by Palanski and Khosla [48]. The latter researchers, however, proposed a modified hypothesis, which states that cystamine forms mixed disulphides with a triad of cysteinyl residues on the surface of transglutaminase 2 that regulates the activation of the extracellular pool of this enzyme. According to Palanski and Khosla [48], cystamine reacts with Cys^230^, Cys^370^, or Cys^371^ to promote the eventual formation of an allosteric disulphide bond between Cys^370^ and Cys^371^ as shown in Figure 1C. These mechanisms, however, presume that cystamine is not metabolized *en route* to the targetted transglutaminases; a presumption that is not supported by pharmacokinetic studies.

## Conversion of cystamine into cysteamine within the body

Cystamine is rapidly reduced to cysteamine by serum, as well as by the liver and kidneys [49]. By contrast, cysteamine is relatively stable in plasma and rapidly absorbed from blood into tissues [49–53]. Prior to cellular uptake, cysteamine undergoes thiol-disulphide interchange with extracellular cystine to form cysteamine–cysteine mixed disulphide (Figure 1D), which resembles lysine [54,55]. Consequently, the cysteamine–cysteine mixed disulphide enters cells through amino acid transporters and is then reduced to cysteamine and cysteine. Thus, the major form in which cystamine inhibits intracellular transglutaminases is cysteamine and not cystamine.

## Cysteamine as an inhibitor of intracellular transglutaminases

In earlier studies, we demonstrated that cysteamine acts as a substrate for transglutaminase 2 to link this compound to glutaminyl residues by way of an isopeptide linkage forming *N-*(γ-glutamyl)cysteamine (Figure 1E) [56]. In other words, cysteamine by virtue of being a transglutaminase 2 substrate, acts as a competitive inhibitor of the other amine substrates of this enzyme. Cystamine has not been shown to be an amine substrate of transglutaminase 2, an assertion erroneously attributed to us elsewhere [48]. Formation of *N-*(γ-glutamyl)cysteamine by transglutaminases could account for two puzzling observations pertaining to the metabolism of exogenously supplied cysteamine. The first of these observations is that a significant portion of the administered cysteamine is unaccounted for following analysis of established routes of metabolism. Cysteamine generated endogenously by the catabolism of pantetheine is oxidized to hypotaurine and then taurine [57]. The administration of cysteamine to rodents, however, does not result in significant accumulation of hypotaurine or taurine in brain or plasma [49], and indicates that the metabolism of exogenous cysteamine bypasses oxidation to taurine. A small portion of cysteamine administered *per os* is metabolized to thialysine and then *S*-(2-aminoethyl)l-cysteine ketimine decarboxylated dimer [50]. Based on the levels of cysteine that accompany cysteamine into cells as a mixed disulphide, significant quantities of cysteamine must enter cells [38,49–51,54,55] but it is then rapidly metabolized. The cellular fate of the majority of exogenous cysteamine remains unaccounted for.

## A role for transglutaminases in the metabolism of cysteamine

A novel hypothesis for the metabolism of cysteamine is that it is covalently attached to proteins by intracellular transglutaminases. This hypothesis is supported by the observation that a significant portion of radiolabeled cysteamine administered to animals or cells is covalently bound to proteins, but not by disulphide bonds [58,59]. This hypothesis requires that the intracellular transglutaminases be activated while cysteamine is being absorbed by cells. Transglutaminases are activated by calcium. Exogenous cysteamine may stimulate calcium release from intracellular stores and thereby promote transglutaminase activity. This mechanism depends on the production of hydrogen peroxide (*H_2_O_2_*), by micromolar amounts of cysteamine. At these concentrations, thiols (*RSH*) such as cysteamine reduce transition metals (*M^n^* → *M^n^*^−1^, where *n* is the oxidation number), while being oxidized to the corresponding disulphide (*RSSR*):

$$2RSH\;+\;2M^{n}\;⇄\;RSSR\;+\;2M^{n-1}+2H^{+}$$

The reduced metals, in turn, reduce oxygen (*O*_2_) to superoxide ($O_{\dot{2}}^{-}$):

$$M^{n-1}+O_{2}⇄M^{n}+O_{\dot{2}}^{-}$$

Dismutation of superoxide yields hydrogen peroxide (*H*_2_*O*_2_), which is a mild oxidant at physiological pH values (7.2–7.4):

$$2O_{\dot{2}}^{-}\;+\;2H^{+}\;\to \;H_{2}O_{2}\;+\;O_{2}$$

Thiols, such as cysteamine, react slowly with hydrogen peroxide under these conditions [60]. Effective scavenging of the peroxide by thiols does not occur until the thiols are present at millimolar concentrations [54]. Thus, at the micromolar concentrations that it attains outside of cells, cysteamine promotes hydrogen peroxide production by the reactions shown above [54]. Hydrogen peroxide readily enters cells and causes a peroxidative stress that is exacerbated by the inhibition of cellular glutathione peroxidases by cysteamine [54].

Hydrogen peroxide promotes the release of calcium from intracellular stores [61,62] and should therefore stimulate transglutaminase activity. In support of this notion, the addition of hydrogen peroxide to cells in culture stimulates their *in situ* transglutaminase activity [63,64].

The above conjecture could be readily tested by investigating the plasma of cysteamine-treated animals or medium of cells in culture treated with cysteamine for the presence of free *N-*(γ-glutamyl)cysteamine. Isopeptide linkages are resistant to proteolysis and consequently transglutaminase-made *N-*(γ-glutamyl)amines are excised as free *N-*(γ-glutamyl)amines during proteolysis of proteins bearing these species [65]. Free *N-*(γ-glutamyl)amines are present in various body fluids and reflect the levels of active transglutaminases in tissues [3,66,67]. If our hypothesis is correct, then the simultaneous measurements of taurine, *S*-(2-aminoethyl)l-cysteine ketimine decarboxylated dimer, as well as protein-bound and free *N-*(γ-glutamyl)cysteamine should provide a comprehensive accounting of the metabolism of exogenous cyst(e)amine, in addition to indicating the mechanism by which cysteamine inhibits intracellular transglutaminases.

## Sites for the oxidative inactivation of transglutaminases by cystamine

Transglutaminases are fully activated by the binding of three calcium ions per enzyme and reducing conditions that maintain the active site cysteine in a fully reduced state [1,2,47]. The cytosol is highly reducing and therefore the activation of intracellular transglutaminases is regulated by the availability of cytosolic calcium. The extracellular environment is different; calcium is readily available whereas reductants are not. Khosla et al. discovered that the activity of extracellular transglutaminase 2 is regulated by the redox status of two vicinal cysteinyl residues on the surface of this enzyme [68]. Under the oxidizing conditions of interstitial fluids [69], these residues: Cys^370^ or Cys^371^ form a disulphide in a manner that involves a third cysteinyl residue, Cys^230^, and ERp57 [70]. Reduction in the Cys^370^–Cys^371^ disulphide linkage by thioredoxin activates extracellular transglutaminase 2 [71,72]. The activation of extracellular transglutaminase by this mechanism is blocked by cystamine forming mixed disulphides with Cys^370^ and Cys^371^ (Figure 1C). As noted earlier, cystamine is converted into cysteamine in the body [49]. It is possible that a portion of the plasma-derived cysteamine is oxidized to cystamine within the interstitial spaces and in this form inactivates extracellular transglutaminases. The amount of cystamine available to inhibit the extracellular transglutaminases by this mechanism will depend on the amounts of cysteamine and cysteamine–cysteine mixed disulphide; the amounts of the latter are expected to be significant after the administration of cyst(e)amine. It should be noted that cysteamine–cysteine mixed disulphide could also inhibit transglutaminase in an oxidative manner, as shown in Figure 1C with cysteamine–cysteine mixed disulphide replacing cystamine. The lumen of the gut is also likely to be an oxidizing environment because the administration of cysteamine by gavage results in the appearance of cystamine in the plasma [50]; the most likely site for oxidation of cysteamine to cystamine, in this case, is the gut. Thus, cystamine is most likely to inhibit transglutaminases by an oxidative mechanism in the gut. This observation is important since aberrant transglutaminase activities contribute to etiology of several intestinal diseases, in particular, celiac disease. In this disease, transglutaminases act to deamidate glutaminyl residues in the wheat protein gliadin increasing the autoantigenicity of the modified protein in the context of HLA-DQ2 or HLA-DQ8 [73]. Cystamine inhibits the generation of the relevant epitopes *in vitro*, but only at millimolar concentrations [74]. Given the relatively safe use of cysteamine in humans [75–77] and the potential to assess the mechanism by which this compound inhibits transglutaminases (*i.e.*, by measurement of *N-*(γ-glutamyl)cysteamine), cysteamine may be of use in the treatment of celiac disease and other diseases involving transglutaminases.

## Conclusion

The activities of intracellular and extracellular transglutaminases contribute to a number of important pathologies. Agents that safely inhibit the *in situ* activities of these transglutaminase pools are therefore of interest as possible therapeutics. The evidence presented here indicates that cystamine inhibits extracellular transglutaminases, while its reduced congener – cysteamine – inhibits intracellular transglutaminases. This distinction is important for the design of other transglutaminase inhibitors based on the mechanisms by which cysteamine or cystamine inhibit these enzymes (*e.g.*, disulphiram [48]). It may also guide the form in which cystamine is administered: as either cystamine or cysteamine. Finally, the measurement of *N-*(γ-glutamyl)cysteamine) may provide a means of determining the mechanism by which intracellular transglutaminases are inhibited following the administration of cystamine or cysteamine.

## References

[1] Jeitner T.M., Muma N.A., Battaile K.P. and Cooper A.J. (2009) Transglutaminase activation in neurodegenerative diseases. Future Neurol. 4, 449–467 10.2217/fnl.09.17 20161049 PMC2746681

[2] Klöck C. and Khosla C (2012) Regulation of the activities of the mammalian transglutaminase family of enzymes. Protein Sci. 21, 1781–1791 10.1002/pro.2162 23011841 PMC3575910

[3] Nemes Z., Fésüs L., Egerházi A., Keszthelyi A. and Degrell I.M. (2001) N(epsilon)(gamma-glutamyl)lysine in cerebrospinal fluid marks Alzheimer type and vascular dementia. Neurobiol. Aging 22, 403–406 10.1016/S0197-4580(01)00224-X 11378245

[4] Zainelli G.M., Ross C.A., Troncoso J.C. and Muma N.A. (2003) Transglutaminase cross-links in intranuclear inclusions in Huntington disease. J. Neuropathol. Exp. Neurol. 62, 14–24 10.1093/jnen/62.1.14 12528814

[5] Andringa G. (2004) Tissue transglutaminase catalyzes the formation of alpha-synuclein crosslinks in Parkinson’s disease. FASEB J. 18, 932–934 10.1096/fj.03-0829fje 15001552

[6] Zhang J. (2016) Tissue transglutaminase and its product isopeptide are increased in Alzheimer’s disease and appswe/ps1de9 double transgenic mice brains. Mol. Neurobiol. 53, 5066–5078 10.1007/s12035-015-9413-x 26386840 PMC4799778

[7] Halverson R.A., Lewis J., Frausto S., Hutton M. and Muma N.A. (2005) Tau protein is cross-linked by transglutaminase in P301L tau transgenic mice. J. Neurosci. 25, 1226–1233 10.1523/JNEUROSCI.3263-04.2005 15689560 PMC6725970

[8] Junn E., Ronchetti R.D., Quezado M.M., Kim S.Y. and Mouradian M.M. (2003) Tissue transglutaminase-induced aggregation of alpha-synuclein: Implications for Lewy body formation in Parkinson’s disease and dementia with Lewy bodies. Proc. Natl Acad. Sci. U.S.A. 100, 2047–2052 10.1073/pnas.0438021100 12576551 PMC149956

[9] Lesort M., Chun W., Johnson G.V. and Ferrante R.J. (1999) Tissue transglutaminase is increased in Huntington’s disease brain. J. Neurochem. 73, 2018–2027 10537061

[10] Karpuj M.V. (1999) Transglutaminase aggregates huntingtin into nonamyloidogenic polymers, and its enzymatic activity increases in Huntington’s disease brain nuclei. Proc. Natl Acad. Sci. U.S.A. 96, 7388–7393 10.1073/pnas.96.13.7388 10377424 PMC22095

[11] Karpuj M.V., Becher M.W. and Steinman L (2002) Evidence for a role for transglutaminase in Huntington’s disease and the potential therapeutic implications. Neurochem. Int. 40, 31–36 10.1016/S0197-0186(01)00060-2 11738470

[12] Wilhelmus M.M. (2009) Transglutaminases and transglutaminase-catalyzed cross-links colocalize with the pathological lesions in Alzheimer’s disease brain. Brain Pathol. 19, 612–622 10.1111/j.1750-3639.2008.00197.x 18673368 PMC8094859

[13] Luo R. (2016) Transglutaminase is a critical link between inflammation and hypertension. J. Am. Heart Assoc. 5, 10.1161/JAHA.116.003730 PMC5015405 27364991

[14] Byrnes J.R. and Wolberg A.S. (2016) Newly-recognized roles of factor XIII in thrombosis. Semin. Thromb. Hemost. 42, 445–454 10.1055/s-0036-1571343 27056150 PMC5680043

[15] de Jager M. (2015) Tissue transglutaminase-catalysed cross-linking induces Apolipoprotein E multimers inhibiting Apolipoprotein E’s protective effects towards amyloid-beta-induced toxicity. J. Neurochem. 134, 1116–1128 10.1111/jnc.13203 26088696

[16] de Jager M. (2016) The blood clotting Factor XIIIa forms unique complexes with amyloid-beta (Aβ) and colocalizes with deposited Aβ in cerebral amyloid angiopathy. Neuropathol. Appl. Neurobiol. 42, 255–272 10.1111/nan.12244 25871449

[17] de Jager M. (2013) Tissue transglutaminase colocalizes with extracellular matrix proteins in cerebral amyloid angiopathy. Neurobiol. Aging 34, 1159–1169 10.1016/j.neurobiolaging.2012.10.005 23122413

[18] Chabot N., Moreau S., Mulani A., Moreau P. and Keillor J.W. (2010) Fluorescent probes of tissue transglutaminase reveal its association with arterial stiffening. Chem. Biol. 17, 1143–1150 10.1016/j.chembiol.2010.06.019 21035737

[19] Matlung H.L. (2009) Calcification locates to transglutaminases in advanced human atherosclerotic lesions. Am. J. Pathol. 175, 1374–1379 10.2353/ajpath.2009.090012 19717636 PMC2751534

[20] Cho B.R. (2008) Increased tissue transglutaminase expression in human atherosclerotic coronary arteries. Coron. Artery Dis. 19, 459–468 10.1097/MCA.0b013e3283108fc3 18923241

[21] Mastroberardino P.G. (2002) ‘Tissue’ transglutaminase ablation reduces neuronal death and prolongs survival in a mouse model of Huntington’s disease. Cell Death Differ. 9, 873–880 10.1038/sj.cdd.4401093 12181738

[22] Mattheij N.J. (2016) Coated platelets function in platelet-dependent fibrin formation via integrin αIIbβ3 and transglutaminase factor XIII. Haematologica 101, 427–436 10.3324/haematol.2015.131441 26721892 PMC5004391

[23] Beazley K.E., Reckard S., Nurminsky D., Lima F. and Nurminskaya M. (2013) Two sides of MGP null arterial disease: chondrogenic lesions dependent on transglutaminase 2 and elastin fragmentation associated with induction of adipsin. J. Biol. Chem. 288, 31400–31408 10.1074/jbc.M113.495556 24036114 PMC3829453

[24] Matlung H.L. (2012) Transglutaminase activity regulates atherosclerotic plaque composition at locations exposed to oscillatory shear stress. Atherosclerosis 224, 355–362 10.1016/j.atherosclerosis.2012.07.044 22921425

[25] Williams H. (2010) Effect of transglutaminase 2 (TG2) deficiency on atherosclerotic plaque stability in the apolipoprotein E deficient mouse. Atherosclerosis 210, 94–99 10.1016/j.atherosclerosis.2009.11.014 20003977 PMC2874840

[26] Pistea A. (2008) Small artery remodeling and erythrocyte deformability in L-NAME-induced hypertension: role of transglutaminases. J. Vasc. Res. 45, 10–18 10.1159/000109073 17898543

[27] Klöck C., Herrera Z., Albertelli M. and Khosla C. (2014) Discovery of potent and specific dihydroisoxazole inhibitors of human transglutaminase 2. J. Med. Chem. 57, 9042–9064 10.1021/jm501145a 25333388 PMC4234452

[28] Keillor J.W., Apperley K.Y. and Akbar A (2015) Inhibitors of tissue transglutaminase. Trends Pharmacol. Sci. 36, 32–40 10.1016/j.tips.2014.10.014 25500711

[29] McConoughey S.J. (2010) Inhibition of transglutaminase 2 mitigates transcriptional dysregulation in models of Huntington disease. EMBO Mol. Med. 2, 349–370 10.1002/emmm.201000084 20665636 PMC3068019

[30] Oh Y.J. (2017) Role of tissue transglutaminase in age-associated ventricular stiffness. Amino Acids 49, 695–704 10.1007/s00726-016-2295-z 27438265

[31] Lin Y., He H., Luo Y., Zhu T. and Duan R. (2015) Inhibition of transglutaminase exacerbates polyglutamine-induced neurotoxicity by increasing the aggregation of mutant ataxin-3 in an SCA3 *Drosophila* model. Neurotox. Res. 27, 259–267 10.1007/s12640-014-9506-8 25501875

[32] Shin S. (2013) Transglutaminase type 2 in human abdominal aortic aneurysm is a potential factor in the stabilization of extracellular matrix. J. Vasc. Surg. 57, 1362–1370 10.1016/j.jvs.2012.09.062 23538006

[33] Tzang B.S. (2013) Cystamine ameliorates ventricular hypertrophy associated with modulation of IL-6-mediated signaling in lupus-prone mice. Life Sci. 92, 719–726 10.1016/j.lfs.2013.01.027 23399703

[34] Engholm M., Eftekhari A., Chwatko G., Bald E. and Mulvany M.J. (2011) Effect of cystamine on blood pressure and vascular characteristics in spontaneously hypertensive rats. J. Vasc. Res. 48, 476–484 10.1159/000327773 21778764

[35] Hwang I.K. (2009) Expression of tissue-type transglutaminase (tTG) and the effect of tTG inhibitor on the hippocampal CA1 region after transient ischemia in gerbils. Brain Res. 1263, 134–142 10.1016/j.brainres.2009.01.038 19368835

[36] Eftekhari A. (2007) Chronic cystamine treatment inhibits small artery remodelling in rats. J. Vasc. Res. 44, 471–482 10.1159/000106465 17657163

[37] Wang X. (2005) Cerebral PET imaging and histological evidence of transglutaminase inhibitor cystamine induced neuroprotection in transgenic R6/2 mouse model of Huntington’s disease. J. Neurol. Sci. 231, 57–66 10.1016/j.jns.2004.12.011 15792822

[38] Fox J.H. (2004) Cystamine increases L-cysteine levels in Huntington’s disease transgenic mouse brain and in a PC12 model of polyglutamine aggregation. J. Neurochem. 91, 413–422 10.1111/j.1471-4159.2004.02726.x 15447674

[39] Dedeoglu A. (2002) Therapeutic effects of cystamine in a murine model of Huntington’s disease. J. Neurosci. 22, 8942–8950 10.1523/JNEUROSCI.22-20-08942.2002 12388601 PMC6757687

[40] Karpuj M.V. (2002) Prolonged survival and decreased abnormal movements in transgenic model of Huntington disease, with administration of the transglutaminase inhibitor cystamine. Nat. Med. 8, 143–149 10.1038/nm0202-143 11821898

[41] Van Raamsdonk J.M. (2005) Cystamine treatment is neuroprotective in the YAC128 mouse model of Huntington disease. J. Neurochem. 95, 210–220 10.1111/j.1471-4159.2005.03357.x 16181425

[42] Gibrat C. and Cicchetti F (2011) Potential of cystamine and cysteamine in the treatment of neurodegenerative diseases. Prog. Neuropsychopharmacol. Biol. Psychiatry 35, 380–389 10.1016/j.pnpbp.2010.11.023 21111020

[43] Sun L. (2010) Effects of cysteamine on MPTP-induced dopaminergic neurodegeneration in mice. Brain Res. 1335, 74–82 10.1016/j.brainres.2010.03.079 20380823

[44] Dubinsky R. and Gray C. (2006) CYTE-I-HD: phase I dose finding and tolerability study of cysteamine (Cystagon) in Huntington’s disease. Mov. Disord. 21, 530–533 10.1002/mds.20756 16258942

[45] Prundean A., Youssov K., Humbert S., Bonneau D. and Verny C (2015) A phase II, open-label evaluation of cysteamine tolerability in patients with Huntington’s disease. Mov. Disord. 30, 288–289 10.1002/mds.26101 25475049

[46] Lorand L. and Conrad S.M. (1984) Transglutaminases. Mol. Cell. Biochem. 58, 9–35 10.1007/BF00240602 6143256

[47] Jeon J.H. (2004) Different inhibition characteristics of intracellular transglutaminase activity by cystamine and cysteamine. Exp. Mol. Med. 36, 576–581 10.1038/emm.2004.74 15675041

[48] Palanski B.A. and Khosla C. (2018) Cystamine and disulfiram inhibit human transglutaminase 2 via an oxidative mechanism. Biochemistry, 10.1021/acs.biochem.8b00204 29570977 PMC6008213

[49] Pinto J.T. (2005) Treatment of YAC128 mice and their wild-type littermates with cystamine does not lead to its accumulation in plasma or brain: implications for the treatment of Huntington disease. J. Neurochem. 94, 1087–1101 10.1111/j.1471-4159.2005.03255.x 15992377

[50] Pinto J.T. (2009) Measurement of sulfur-containing compounds involved in the metabolism and transport of cysteamine and cystamine. Regional differences in cerebral metabolism. J. Chromatogr. B Analyt. Technol. Biomed. Life Sci. 877, 3434–3441 10.1016/j.jchromb.2009.05.041 19523884 PMC2752955

[51] Bousquet M. (2010) Cystamine metabolism and brain transport properties: clinical implications for neurodegenerative diseases. J. Neurochem. 114, 1651–1658 10.1111/j.1471-4159.2010.06874.x 20569301

[52] Dohil R., Cabrera B.L., Gangoiti J.A., Barshop B.A. and Rioux P. (2014) Pharmacokinetics of cysteamine bitartrate following intraduodenal delivery. Fundam. Clin. Pharmacol. 28, 136–143 10.1111/fcp.12009 23113697

[53] Bouazza N. (2011) Population pharmacokinetics and pharmacodynamics of cysteamine in nephropathic cystinosis patients. Orphanet J. Rare Dis. 6, 86 10.1186/1750-1172-6-86 22195601 PMC3257201

[54] Jeitner T.M. and Lawrence D.A. (2001) Mechanisms for the cytotoxicity of cysteamine. Toxicol. Sci. 63, 57–64 10.1093/toxsci/63.1.57 11509744

[55] Meier T. and Issels R.D. (1995) Promotion of cyst(e)ine uptake. Methods Enzymol. 252, 103–112 10.1016/0076-6879(95)52013-9 7476343

[56] Jeitner T.M., Delikatny E.J., Ahlqvist J., Capper H. and Cooper A.J. (2005) Mechanism for the inhibition of transglutaminase 2 by cystamine. Biochem. Pharmacol. 69, 961–970 10.1016/j.bcp.2004.12.011 15748707

[57] Jacobsen J.G. and Smith L.H. (1968) Biochemistry and physiology of taurine and taurine derivatives. Physiol. Rev. 48, 424–511 10.1152/physrev.1968.48.2.424 4297098

[58] Modig H.G., Edgren M. and Révész L (1972) Release of thiols from cellular mixed disulphides and its possible role in radiation protection. Int. J. Radiat. Biol. Relat. Stud. Phys. Chem. Med. 22, 257–268 10.1080/09553007214551031 4343885

[59] Power J.A., Goldstein L.S. and Harris J.W. (1974) Letter: a test of the ‘mixed-disulphide’ hypothesis of cysteamine radioprotection. Int. J. Radiat. Biol. Relat. Stud. Phys. Chem. Med. 26, 91–96 10.1080/09553007414551011 4608036

[60] Winterbourn C.C. and Metodiewa D (1999) Reactivity of biologically important thiol compounds with superoxide and hydrogen peroxide. Free Radic. Biol. Med. 27, 322–328 10.1016/S0891-5849(99)00051-9 10468205

[61] Gibson G.E., Zhang H., Xu H., Park L.C. and Jeitner T.M. (2002) Oxidative stress increases internal calcium stores and reduces a key mitochondrial enzyme. Biochim. Biophys. Acta 1586, 177–189 10.1016/S0925-4439(01)00091-6 11959459

[62] Wang X., Takeda S., Mochizuki S., Jindal R. and Dhalla N.S. (1999) Mechanisms of hydrogen peroxide-induced increase in intracellular calcium in cardiomyocytes. J. Cardiovasc. Pharmacol. Ther. 4, 41–48 10.1177/107424849900400107 10684523

[63] Lee Z.W. (2003) Activation of *in situ* tissue transglutaminase by intracellular reactive oxygen species. Biochem. Biophys. Res. Commun. 305, 633–640 10.1016/S0006-291X(03)00835-0 12763041

[64] Yi S.J. (2004) Arachidonic acid activates tissue transglutaminase and stress fiber formation via intracellular reactive oxygen species. Biochem. Biophys. Res. Commun. 325, 819–826 10.1016/j.bbrc.2004.10.122 15541364

[65] Jeitner T.M., Battaile K. and Cooper A.J. (2013) γ-Glutamylamines and neurodegenerative diseases. Amino Acids 44, 129–142 10.1007/s00726-011-1209-3 22407484 PMC3491119

[66] Jeitner T.M. (2001) N(epsilon)-(gamma-L-glutamyl)-L-lysine (GGEL) is increased in cerebrospinal fluid of patients with Huntington’s disease. J. Neurochem. 79, 1109–1112 10.1046/j.1471-4159.2001.00673.x 11739625

[67] Jeitner T.M., Matson W.R., Folk J.E. and Blass J.P. Cooper A.J. (2008) Increased levels of gamma-glutamylamines in Huntington disease CSF. J. Neurochem. 106, 37–44 10.1111/j.1471-4159.2008.05350.x 18422943 PMC2574808

[68] Stamnaes J., Pinkas D.M., Fleckenstein B., Khosla C. and Sollid L.M. (2010) Redox regulation of transglutaminase 2 activity. J. Biol. Chem. 285, 25402–25409 10.1074/jbc.M109.097162 20547769 PMC2919103

[69] Yi M.C. and Khosla C. (2016) Thiol-disulfide exchange reactions in the mammalian extracellular environment. Annu. Rev. Chem. Biomol. Eng. 7, 197–222 10.1146/annurev-chembioeng-080615-033553 27023663 PMC4899241

[70] Yi M.C., Melkonian A.V., Ousey J.A. and Khosla C. (2018) Endoplasmic reticulum-resident protein 57 (ERp57) oxidatively inactivates human transglutaminase 2. J. Biol. Chem. 293, 2640–2649 10.1074/jbc.RA117.001382 29305423 PMC5827427

[71] Plugis N.M., Palanski B.A., Weng C.H., Albertelli M. and Khosla C. (2017) Thioredoxin-1 selectively activates transglutaminase 2 in the extracellular matrix of the small intestine: implications for celiac disease. J. Biol. Chem. 292, 2000–2008 10.1074/jbc.M116.767988 28003361 PMC5290969

[72] Jin X. (2011) Activation of extracellular transglutaminase 2 by thioredoxin. J. Biol. Chem. 286, 37866–37873 10.1074/jbc.M111.287490 21908620 PMC3199528

[73] Hadjivassiliou M. (2010) Gluten sensitivity: from gut to brain. Lancet Neurol. 9, 318–330 10.1016/S1474-4422(09)70290-X 20170845

[74] Molberg O. (2001) T cells from celiac disease lesions recognize gliadin epitopes deamidated in situ by endogenous tissue transglutaminase. Eur. J. Immunol. 31, 1317–1323 10.1002/1521-4141(200105)31:5%3c1317::AID-IMMU1317%3e3.0.CO;2-I 11465088

[75] Langman C.B. (2012) A randomized controlled crossover trial with delayed-release cysteamine bitartrate in nephropathic cystinosis: effectiveness on white blood cell cystine levels and comparison of safety. Clin. J. Am. Soc. Nephrol. 7, 1112–1120 10.2215/CJN.12321211 22554716 PMC3386675

[76] Dohil R. and Cabrera B.L. (2013) Treatment of cystinosis with delayed-release cysteamine: 6-year follow-up. Pediatr. Nephrol. 28, 507–510 10.1007/s00467-012-2315-5 23001048

[77] Medic G., van der Weijden M., Karabis A. and Hemels M. (2017) A systematic literature review of cysteamine bitartrate in the treatment of nephropathic cystinosis. Curr. Med. Res. Opin. 33, 2065–2076 10.1080/03007995.2017.1354288 28692321

